# The Combination of *Astragalus membranaceus* and *Angelica sinensis* Inhibits Lung Cancer and Cachexia through Its Immunomodulatory Function

**DOI:** 10.1155/2019/9206951

**Published:** 2019-11-03

**Authors:** Tsung-Han Wu, Kun-Yun Yeh, Chen-Hsu Wang, Hang Wang, Tsung-Lin Li, Yi-Lin Chan, Chang-Jer Wu

**Affiliations:** ^1^Department of Food Science and Center of Excellence for the Oceans, National Taiwan Ocean University, Keelung 20224, Taiwan; ^2^Division of Hemato-Oncology, Department of Internal Medicine, Chang Gung Memorial Hospital, Keelung 20401, Taiwan; ^3^Chang Gung University College of Medicine, Taoyuan 33302, Taiwan; ^4^Institute of Biomedical Nutrition, Hung-Kuang University, Taichung 43302, Taiwan; ^5^Genomics Research Center, Academia Sinica, Taipei 11574, Taiwan; ^6^Department of Life Science, Chinese Culture University, Taipei 11114, Taiwan; ^7^Department of Medical Research, China Medical University Hospital, China Medical University, Taichung 40402, Taiwan; ^8^Department of Health and Nutrition Biotechnology, Asia University, Taichung 41354, Taiwan; ^9^Graduate Institute of Medicine, Kaohsiung Medical University, Kaohsiung, Taiwan

## Abstract

Lung cancer and its related cachexia are the leading cause of cancer death in the world. In this study, we report the inhibitory effect of the combined therapy of *Astragalus membranaceus* and *Angelica sinensis*, on tumor growth and cachexia in tumor-bearing mice. Lewis lung carcinoma cells were inoculated into male C57BL/6 and CAnN.Cg-Foxn1^nu^ nude mice. After tumor inoculation, mice were fed orally by the combination of AM and AS in different doses. In C57BL/6 mice, the combination of AM and AS significantly inhibited the growth of cancer tumor and prevented the loss of body weight and skeletal muscle. It also diminished the formation of free radicals and cytokines, stimulated the differentiation of NK and Tc cells, and rebalanced the ratios of Th/Tc cells, Th1/Th2 cytokines, and M1/M2 tumor-associated macrophages. The herbal combination also downregulated the expression of NF*κ*Β, STAT3, HIF-1*α*, and VEGF in tumors. In contrast, the findings were not observed in the nude mice. Therefore, the combination of AM and AS is confirmed to inhibit the progression of lung cancer, cancer cachexia, and cancer inflammation through the immunomodulatory function.

## 1. Introduction

Lung cancer is the leading cause of cancer death in the world. In United States, 234,030 new cases are estimated to be diagnosed, and 154,050 disease-related deaths are estimated to occur in 2008 [1]. In Taiwan, 12,462 new cases can be diagnosed annually and 9,167 people may die from the disease every year [2]. Although many improvements have been made recently such as minimally invasive operation and immunotherapy, only 18% of the patients live 5 years or more after diagnosis [3].

Cachexia is a common symptom in patients with lung cancer [3]. It is a characteristic of unrecoverable fatigue and involuntary loss of appetite, body weight, skeletal muscle, and fat content [3–5]. Cachexia tremendously affects the outcome of patients with lung cancer. In a Japanese study, the median survival of cachectic patients reduced nearly 1.5 years compared to that of noncachectic patients [3]. These facts indicate a vital and constant need to develop new treatment methods overcoming lung cancer and its cachexia.

Tumor-associated macrophages (TAMs) are macrophages within tumor microenvironment. Similar to other macrophages, TAMs can be an activated M1 phenotype characterized by the expression of iNOS, IL-1*β*, IL-6, tumor necrosis factor-*α* (TNF-*α*), CCL2, ROS, and NO. Alternatively, TAMs can present an immunosuppressive M2 phenotype, which was characterized by the expression of arginase 1, CD163, IL-10, Ym1, CD36, CD204, and CD206 [6–12]. Meanwhile, the M1-phenotype TAMs can be activated by cytokines of the T helper 1 (Th1) immune responses, such as interferon-*γ* (INF-*γ*) and TNF-*α*. In contrast, M2 TAMs are activated by the Th2 cytokines like IL-4 and IL-10 and responsible to the expression of IL-10 and arginase, immunosuppression, as well as the promotion of tumor growth [6–10]. In clinical researches, TAMs and Th1/Th2 cytokines have been connected to treatment response and survival time in patients with lung cancer [6, 7]. Taking together, these findings indicate a close relationship between cancer, macrophages, and cytokines.

TAMs and Th1/Th2 cytokines are also correlated to cancer cachexia [9, 10, 13, 14]. Smoldering and continuous inflammation have been observed in patients with cancer cachexia [14]. The increasing serum levels of reactive oxygen species (ROS), nitric oxide (NO), inflammatory cytokines (e.g., IL-1*β*, IL-4, IL-6, and TNF-*α*), as well as the upregulation of nuclear factor kappa-light-chain-enhancer of activated B cells (NF-*κ*B) and signal transducer and activator of transcription 3 (STAT3), have been discovered in patients and animals with lung cancers [6, 7, 9, 10]. These inflammatory cytokines, including IL-6, are generally produced by macrophages. After activation by IL-6, STAT3 can stimulate myeloid cells differentiate into TAMs [14]. In brief, TAMs and these cytokines play an important character in the development and maintenance of cancer cachexia.

*Astragalus membranaceus* (AM) and *Angelica sinensis* (AS) are two major herbs applied by Chinese for hundred years. Traditionally, they are used as tonic drugs to treat fatigue, dizziness, anemia, shortness of breath, and gynecologic disorders [15]. Modern research studies have mentioned the potential benefits of AM and AS to patients with lung cancer and cachexia. AM, AS, and their ingredients, such as astragalus polysaccharides (APS), astragalus saponin (AST), astragaloside IV (AS-IV), calycosin, ferulic acid, formononetin, and Z-ligustilide (LIG), were proved to suppress the growth of tumor cells, the release of inflammatory cytokines, the expression of NF-*κ*B and STAT3, and the activation of protumoral macrophages and T cells [8, 16–23]. Recently, the combination therapy of AM and AS, especially in a weight ratio of 5 : 1 (a.k.a. Danggui Buxue Tang (DBT)), has been proved to have anticancer and anticachectic effects [24, 25]. Therefore, we designed the following experiments to verify our hypothesis that DBT has better anticancer, anti-inflammatory, and anticachectic effects as compared to AM or AS.

## 2. Materials and Methods

### 2.1. Preparation of Herbal Extracts

The aqueous extracts of AM and AS were kindly provided by Chuang Song Zong Pharmaceutical Co. Ltd. (Kaohsiung, Taiwan), with has a certification of Good Manufacturing Practice. The raw plant materials of AM and AS were obtained from Inner Mongolia and Gansu Province, China, respectively. The identification and authentication of herbs were arranged by qualified experts of the company. The extractions of AM and AS were prepared by boiling in 10-fold (w/w) of water for 90 minutes. All water extractions were concentrated by lyophilization. The combination of AM and AS (DBT) was prepared by mixing the extracts of AM and AS in a 5 : 1 weight ratio.

### 2.2. Phytochemical Analysis of Herbal Extracts

Ultraperformance liquid chromatography (UPLC) fingerprint was applied to the authentication of AM and AS extracts. Formononetin (purity: 98.43%, Tauto, Shanghai, China), calycosin-7-glucoside (purity: 98.3%, National Institutes for Food and Drug Control, Beijing, China), and AS-IV (purity: 98.3%, Must, Chengdu, China) were used as the reference standards of AM. In addition, ferulic acid (purity: 100%, Sigma-Aldrich, St. Louis, MO, USA) was used as the reference standard of AS. The chromatography was performed in the Acquity UPLC PDA/ELSD *eλ* detector (Waters, Milford, MA, USA). For the determination of AS-IV, the Acquity UPLC HSS C18 column, 2.1 × 100 mm, 1.8 *μ*m (Waters, Milford, MA, USA), was selected. The column temperature was set at 30°C. The injection volume was 3 *μ*l. The gradient solution was 40% (v/v) acetonitrile in pure water. The flow rate was 0.3 ml/min. The ELSD drift tube temperature was 50°C, and the pressure was set at 20 psi. For the determination of formononetin and calycosin-7-glucoside, the column, the column temperature, and the injection volume were as same as those of AS-IV. The gradient solution A was 0.2% (v/v) formic acid in pure water, and acetonitrile was used as solution B. The gradient program was as follows: 0–23 min, from 95% A and 5% B to 55% A and 45% B. The flow rate was set at 0.4 ml/min. The detection wavelength was 254 nm. For the determination of ferulic acid, the Acquity UPLC HSS T18 column, 2.1 × 100 mm, 1.8 *μ*m (Waters, Milford, MA, USA), was set at a temperature of 35°C. The injection volume was 3 *μ*l. The gradient solution A was 0.1% (v/v) phosphoric acid in pure water. The gradient solution B was 0.1% (v/v) phosphoric acid in acetonitrile/methanol (7 : 3). The gradient program was as follows: 0–28 min, from 90% A and 10% B to 50% A and 50% B. The flow rate was set at 0.3 ml/min. The detection wavelength was 270 nm.

### 2.3. Cell Lines and Cell Culture

Lewis lung carcinoma cells (LLC, CRL-1642), murine macrophage RAW264.7 cells (TIB-71), and baby hamster kidney cells (BHK, CCL-10) were obtained from ATCC (Manassas, VA, USA). LLC and RAW264.7 cells were maintained in Dulbecco's modified Eagle's medium (DMEM) supplemented with 10% (v/v) fetal bovine serum (FBS) and antibiotics (100 U/ml penicillin and 100 *μ*g/ml streptomycin) at 37°C in a humid atmosphere with 5% CO_2_. BHK cells were maintained in the RPMI-1640 medium supplemented with 5% FBS and antibiotics (100 U/ml penicillin and 100 *μ*g/ml streptomycin) at 37°C in a humid atmosphere with 5% CO_2_.

### 2.4. Mice and Animal Experiment

Male, 4–6-week-old, weighing 18–22 g, C57BL/6J and CAnN.Cg-Foxn1^nu^ nude mice were obtained from the National Laboratory Animal Center (Taipei, Taiwan). Mice were housed individually in a climate-controlled room of 12 : 12 dark-light cycle with a constant room temperature of 21.6°C. Before treatment, mice acclimated the new environment and food for ≥4 days. Mice could freely intake water and rodent diet (Young Li Co., New Taipei City, Taiwan). The whole animal study included two subgroups of murine experiments, which used C57BL/6J and nude mice, respectively. Both subgroups have the same protocol as follows. Mice were divided into five weight-matched groups (*n* = 10 each). In the control (C) group, 0.1 ml of sterile normal saline was injected subcutaneously in the right thigh of each mouse. In tumor-bearing mice, 5 × 10^5^ LLC cells were diluted in 0.1 ml of sterile normal saline and inoculated into the right thigh of each mouse. The tumor-bearing mice were distributed into four groups: the no treatment (T) group, L group (DBT 1 mg/kg/d, orally administered for 25 days), M group (DBT 2.5 mg/kg/d, orally administered for 25 days), and H group (DBT 5 mg/kg/d, orally administered for 25 days). After 25 days, all mice were sacrificed, and the blood was sampled by cardiac puncture. Meanwhile, the inoculated tumors, gastrocnemius muscle, and spleen were resected for the further examination.

### 2.5. In Vitro Cytotoxicity Examination

The 3-(4,5-dimethylthiazol-2-yl)-5-(3-carboxymethoxyphenyl)-2-(4-sulfophenyl)-2H-tetrazolium (MTS) assay was used to test the cytotoxicity of AM, AS, and DBT in vitro. LLC and BHK cells were seeded (1 × 10^4^ cell/ml) in 96-well culture plate, cultured with three herbal extracts in different concentrations (0.625, 1.25, 2.5, 5, and 10 mg/ml) for 24 h, treated with the CellTiter 96 AQueous MTS reagent (Promega, Madison, WI, USA) 20 *μ*l per well and finally incubated in the 37°C, 5% CO_2_ atmosphere for 1 h. The ratio of cell proliferation was measured by fluorescence at an emission wavelength of 490 nm.

### 2.6. In Vitro Antioxidative Examination

The ethanolic 2,2-diphenyl-1-picrylhydrazyl (DPPH) scavenging, superoxide scavenging, and glutathione (GSH) production assays were used to test the antioxidation ability of herbal extracts in vitro. In the DPPH assay, 100 *μ*l DPPH (0.1 mM) was added to 100 *μ*l herbal extract. The mixing solution was left in the dark at room temperature for 30 min, and then its absorbance was measured at the wavelength of 517 nm. In superoxide scavenging assay, 50 *μ*l herbal extract was treated with 50 *μ*l NBT (300 *μ*M), 50 *μ*l NADH (936 *μ*M), and 50 *μ*l PMS (120 *μ*M). The mixing solution was left at room temperature for 5 min, and its absorbance was measured at the wavelength of 560 nm. In the GSH production assay, RAW264.7 cells (5 × 10^5^ cells/ml) were cultured in 6-well plate, treated with herbal extracts in different concentrations, washed, added with trypsin, centrifugated, treated with the GSH assay buffer and Thiolite Green (AAT Bioquest, Sunnyvale, CA, USA), and left at 37°C for 15 min. The cell accumulation of GSH was observed by flow cytometry at the excitation and emission wavelengths of 488 and 525 nm.

### 2.7. In Vitro Anti-Inflammatory Examination

RAW264.7 cells (5 × 10^5^ cells/ml) were cultured in 6-well plate, treated with LPS (1 *μ*g/ml) and herbal extract in different concentration, and left at room temperature. After 24 h, the suspension was collected to measure the concentrations of cytokines. The enzyme-linked immunosorbent assay (ELISA) and Quantikine ELISA kits (R&D, Minneapolis, MI, USA) were applied to measure the levels of IL-1*β*, IL- 6, and TNF-*α*. The absorbance was measured at the wavelength of 450 nm.

### 2.8. In Vitro Phagocytosis Examination

RAW264.7 cells (5 × 10^5^ cells/ml) were cultured in 96-well plate, treated with total 40 *μ*l LPS (1 *μ*g/ml) and herbal extract in different concentrations, and then incubated at 37°C and 5% CO_2_ atmosphere for 16 h. After incubation, the mixture was added with 200 *μ*l/well neutral red (0.075%), left in the dark site at 37°C and 5% CO_2_ atmosphere for 30 min, treated with 200 *μ*l/well acetic acid/anhydrous alcohol (100 mM), and left in the dark site again for 10 h. The absorbance was measured at the wavelength of 550 nm.

### 2.9. Measurement of Serum Reactive Oxygen Species and NO in Mice

The dichlorofluorescin diacetate (DCFH-DA), dihydroethidium (DHE), and 4,5-diaminofluorescein diacetate (DAF-2/DA) assays were applied to evaluate the production of ROS (H_2_O_2_ and superoxide) and NO in tumor-bearing mice. In the DCFH/DA assay, murine blood was added with 1 ml RBC lysis buffer, centrifuged at 1000 rpm for 5 min, removed the suspension, and then treated with 2 ml PBS and 10 *μ*M DCFH-DA. The mixture was incubated in the dark site for 30 minutes, washed, and then resuspended. The accumulation of oxidized product, 2′,7′-dichlorofluorescein (DCF), was observed by flow cytometry at the excitation and emission wavelengths of 488 and 525 nm. In the DHE assay, murine blood was added with 1 ml RBC lysis buffer, centrifuged at 1000 rpm for 5 min, removed the suspension, and then treated with 2 ml PBS and 10 *μ*M DHE. The mixture was incubated in the dark site for 30 minutes, washed, and then resuspended. The accumulation of oxidized product, 2-hydroxyethidium (2-OH-E+), was observed by flow cytometry at the excitation and emission wavelengths of 488 and 575 nm. In the DAF-2/DA assay, murine blood was added with 1 ml RBC lysis buffer, centrifuged at 1000 rpm for 5 min, removed the suspension, and then treated with 2 ml PBS and 10 *μ*M DAF-2/DA. The mixture was incubated in the dark site for 30 minutes, washed, and then resuspended. The accumulation of the oxidized product, diaminofluorescein-triazole (DAF-2T), was observed by flow cytometry at the excitation and emission wavelengths of 488 and 525 nm.

### 2.10. Measurement of Serum Albumin and Cytokines in Mice

The inflammation of tumor-bearing mice was observed by measuring serum levels of albumin and inflammatory cytokines. Murine blood was collected and centrifuged at 4°C and 3500 rpm for 30 min, and then the suspension was collected to measure the serum levels of albumin and cytokine. The concentration of albumin was assessed by Spotchemez SP-4430 dry chemical system (Arkray, Kyoto, Japan). The ELISA and Quantikine ELISA kits (R&D, Minneapolis, MI, USA) were applied to measure the levels of IL-1*β*, IL-4, IL- 6, TNF-*α*, and IFN-*γ*. The absorbance was measured at the wavelength of 450 nm.

### 2.11. Peripheral Blood Cells and Splenocyte Differentiation Analysis

Murine blood and spleens were collected to evaluate the influence of DBT to peripheral blood cells and splenocyte differentiation. The counts of peripheral blood cells were calculated by using a Sysmex K-1000 automated hematology analyzer (Sysmex American, Lincolnshire, IL, USA). The differentiation of splenocytes was evaluated by flow cytometry. The spleen was treated with 2 ml RBC lysis buffer to isolate splenocytes. The BD FACSCantoTM II system (BD, San Jose, CA, USA) was used for cytometry. Murine anti-CD3*ε* (BD, San Jose, CA, USA), anti-CD4 (BD, San Jose, CA, USA), anti-CD8a (BD, San Jose, CA, USA), anti-CD19 (BD, San Jose, CA, USA), and anti-CD56 (Bioss, Woburn, MA, USA) antibodies were used to label total T, Th, cytotoxic T (Tc), B, and natural killer (NK) cells, respectively. The antibodies were stained by FITC (fluorescein isothiocyanate) and PE (phycoerythrin).

### 2.12. Protein Extraction and Western Blot

The western blot was used to observe the expression of NF-*κ*B, STAT3, hypoxia-inducible factor 1-alpha (HIF-1*α*), and vascular endothelial growth factor (VEGF) in tumor microenvironment. The inoculated tumor was resected and treated with a lysis buffer solution (20 mM Tris-HCl, pH 7.5, 2 mM ATP, 5 mM MgCl_2_, 1 mM dithiothreitol) and protease inhibitor cocktail (Sigma-Aldrich, Saint Louis, MI, USA), in order to obtain tumor proteins. The sodium dodecyl sulfate polyacrylamide gel electrophoresis (SDS-PAGE) was applied for the western blot. Murine anti-NF-*κ*B (Bio-Rad, Hercules, CA, USA), anti-NF-*κ*B p65 phospho S536 (Abcam, Cambridge, UK), anti-STAT3 (Santa Cruz, Dallas, TX, USA), anti-p-STAT3 (Santa Cruz, Dallas, TX, USA), anti-HIF-1*α* (Bioss, Woburn, MA, USA), and anti-VEGF (Bio-Rad, Hercules, CA, USA) antibodies were used to label tumor proteins. The tumor proteins were also treated with the Pierce BCA protein assay kits (Thermo Fischer, Waltham, MA, USA), and their absorbance was measured at the wavelength of 550 nm.

### 2.13. Immunofluorescence Examination

The immunofluorescence (IF) technique was used to detect the M1- and M2-phenotype TAMs in tumor microenvironment. Rabbit anti-mouse iNOS (Bioss, Woburn, MA, USA) and goat anti-mouse arginase 1 (Santa Cruz, Dallas, TX, USA) were used as primary antibodies in order to label M1 and M2 macrophages, respectively. The secondary antibodies included goat anti-rabbit IgG (Bioss, Woburn, MA, USA) and donkey anti-goat IgG (Santa Cruz, Dallas, TX, USA). In the IF examination, tumor specimens were harvested, fixed in 4% formalin for 48 h, embedded in paraffin, sectioned, deparaffinized in xylene, and rehydrated in ethanol. After antigen retrieval by boiling in 10 mM Tri-EDTA (pH 8.0) for 1 h, the sections were washed with PBST (1 × phosphate-buffered saline (PBS), 0.1% Tween 20), incubated with primary antibodies, washed again with PBST, treated with secondary antibodies, and left in the dark site for 1 h. The slides were observed and pictured by an upright fluorescence microscopy (Olympus BX51, Olympus, Tokyo, Japan). The pictured images were analyzed and merged by ImageJ software (NIH, Bethesda, MD, USA). ImageJ with the colocalization and color deconvolution plugins were also used to quantify immunofluorescence and chromogenic signal intensity on image.

### 2.14. Statistical Analysis

IBM SPSS Statistics 20 software (IBM, Armonk, NY, USA) was used for statistical analysis. Data were shown as mean ± standard error of mean (SEM). Difference between groups was assessed by one-way analysis of variance (ANOVA). Statistical significance of difference was considered at *p* < 0.05.

## 3. Results

### 3.1. Phytochemical Characteristics of AM and AS

**Figure 1:**
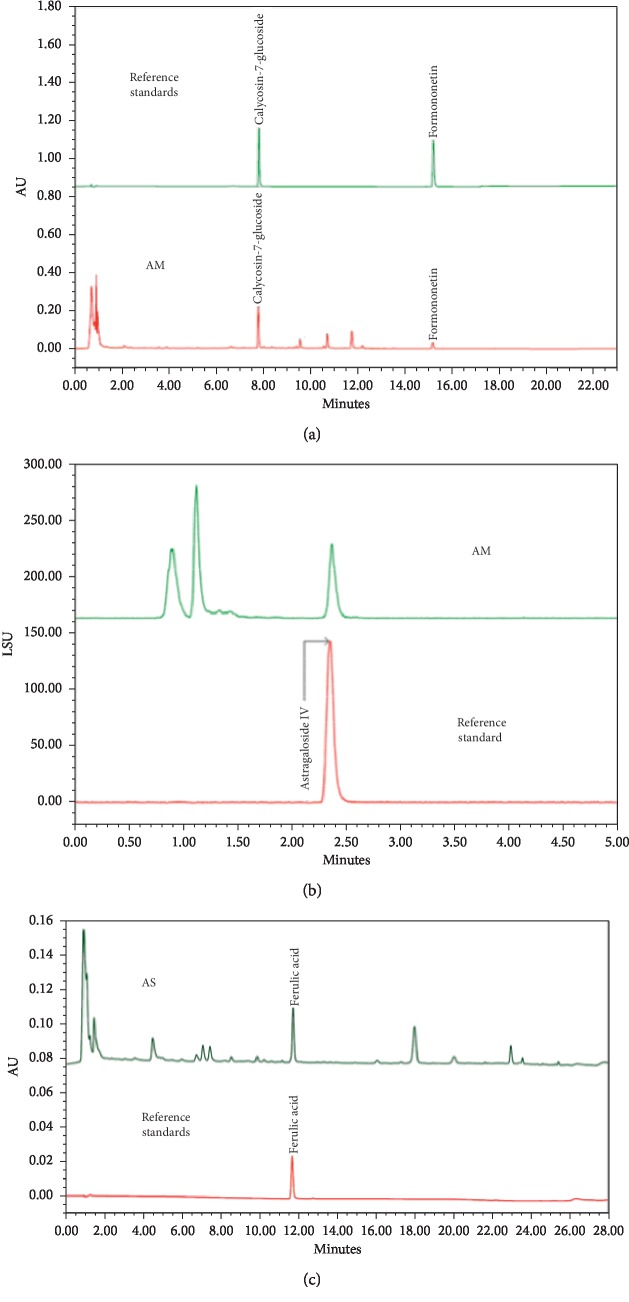
Chromatogram of herbs analyzed using (a) UPLC-PDA and (b) UPLC-ELSD for *Astragalus membranaceus* and (c) UPLC-PDA for *Angelica sinensis*. AM, *Astragalus membranaceus*. AS, *Angelica sinensis*.

The HPLC fingerprints of AM and AS are presented in Figure 1. The reference standards of AM included AS-IV, formononetin, and calycosin-7-glucoside. The reference standard of AS was ferulic acid. These components were confirmed qualitatively and quantitatively in AM and AS. The contents of AS-IV and calycosin-7-glucoside within AM were 0.744 and 0.507 mg/g, respectively. The contents of ferulic acid within AS were 0.733 mg/g. In addition, the contents of AS-IV, calycosin-7-glucoside, and ferulic acid within DBT were 0.62, 0.423, and 0.122 mg/g, respectively.

### 3.2. In Vitro Cytotoxic and Phagocytotic Properties of Herbs

**Figure 2:**
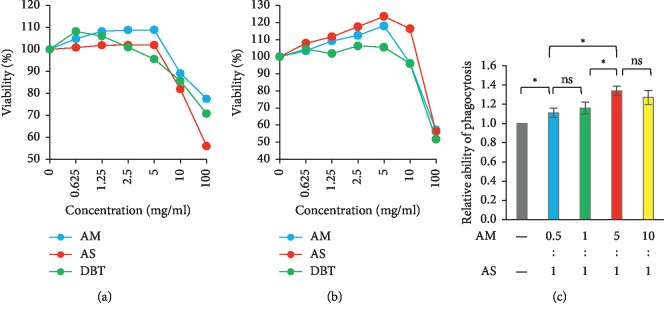
Cytotoxic and phagocytotic effects of *Astragalus membranaceus*, *Angelica sinensis*, and their combination. The MTS assay was used to measure the cytotoxicity to normal BHK (a) and malignant LLC (b) cells. The phagocytotic function was measured by LPS-stimulated RAW264.7 cells (c). AM, *Astragalus membranaceus.* AS, *Angelica sinensis*. DBT, the combination of AM and AS in a weight ratio of 5 : 1. ^*∗*^*p* < 0.05 between two groups. ns, no statistical significance between two groups.

The cytotoxicity of herbal extracts to BHK and LLC cells is presented in Figures 2(a) and 2(b). AM, AS, and DBT have no significant cytotoxicity to normal BHK cells until the concentration was more than 5 mg/ml. In analogy to BHK cells, all three herbs did not cause LLC cell death until the concentration was more than 10 mg/ml. But provided the herbal concentration at 100 mg/ml, the BHK and LLC viabilities were still more than 50%. Given also the fact that the typical concentration of AM or AS is less than 0.3 mg/ml in traditional Chinese medicine, we consider both AM and AS have no cytotoxicity to normal or malignant cells.

As illustrated in Figure 2(c), the phagocytotic effect of LPS-stimulated RAW264.7 cells was enhanced by the combined treatment of AM and AS. Meanwhile, the ability increased in proportion to the content of AM, and it reached the plateau at the 5 : 1 ratio of AM and AS (aka DBT). As compared with AM and AS, the combination treatment can induce the strongest phagocytotic ability in vitro.

### 3.3. In Vitro Anti-Inflammatory and Antioxidative Abilities of Herbs

**Figure 3:**
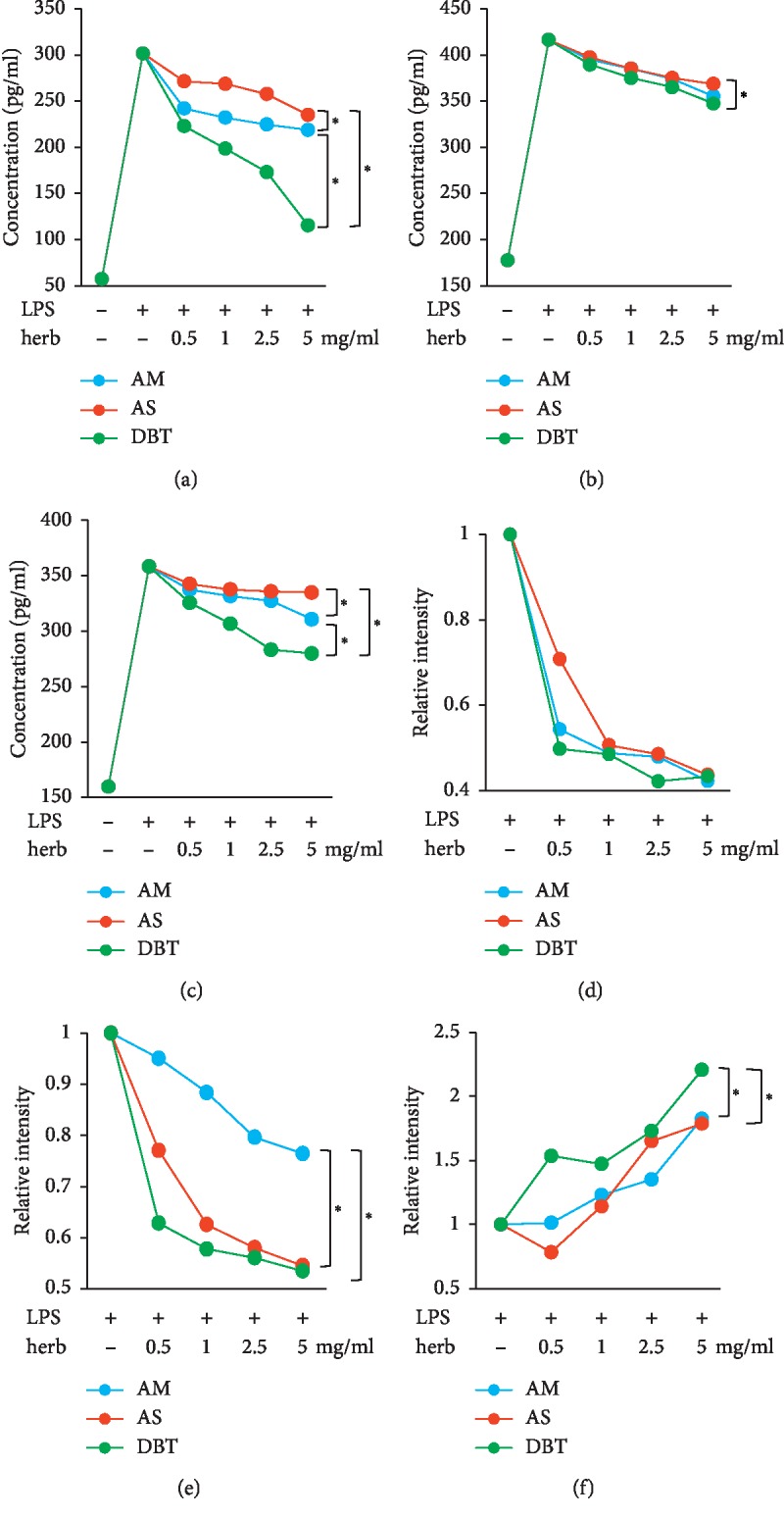
In vitro examinations of anti-inflammatory and antioxidative abilities of *Astragalus membranaceus*, *Angelica sinensis*, and their combination. The anti-inflammatory function was evaluated by measuring the level of IL-1*β* (a), IL-6 (b), and TNF-*α* (c) in LPS-stimulated RAW264.7 cells. The DPPH scavenging (d), superoxide scavenging (e), and GSH production (f) assays were used to measure the antioxidative ability. AM, *Astragalus membranaceus*. AS, *Angelica sinensis*. DBT, the combination of AM and AS in a weight ratio of 5 : 1. ^*∗*^*p* < 0.05 between two groups.

AM, AS, and DBT all exhibited a dose-dependent inhibition to inflammation, as all of them suppressed the generation of IL-1*β*, IL-6, and TNF-*α* in RAW264.7 cells (Figures 3(a)–3(c)). Among the three herbs, DBT was presented as the most effective herb to downregulate these cytokines. In addition, AM, AS, and DBT also represented a dose-dependent efficacy to reduce oxidation (Figures 3(d)–3(f)). The production of H_2_O_2_ and superoxide declined, but the GSH production increased after herbal treatment. Similar to the results of anti-inflammatory experiments, DBT still appeared as the most effective herb to reduce oxidation. These findings indicate that DBT contains anti-inflammatory and antioxidative functions.

### 3.4. In Vivo Anticancer and Anticachectic Abilities of DBT

**Figure 4:**
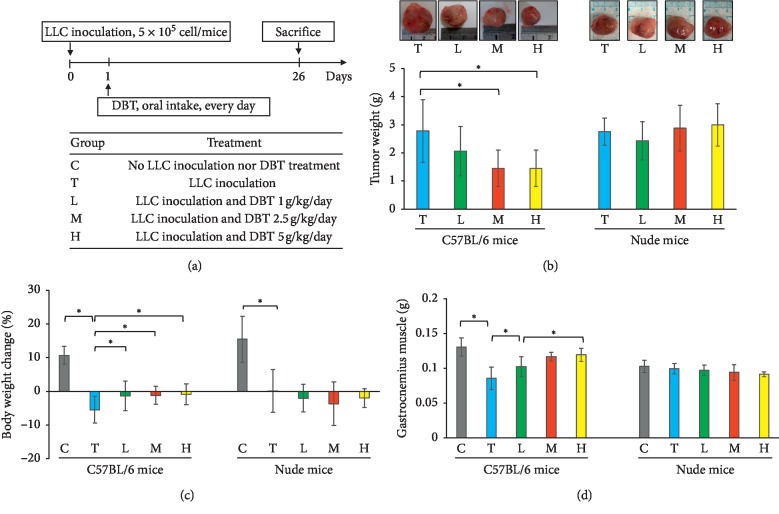
Anticancer and anticachectic effect of the combination of *Astragalus membranaceus* and *Angelica sinensis* in tumor-bearing mice. LLC cells were inoculated in C57BL/6J or CAnN.Cg-Foxn1nu nude mice on day 0. The herbal combination was prescribed from day 1. Sacrifice was arranged on day 26 (a). The inoculated tumors (b), murine body (c), and gastrocnemius muscles (d) were weighed during sacrifice. DBT, the combination of AM and AS in a weight ratio of 5 : 1. C, control group. T, tumor group. L, low-dose treatment group. M, middle-dose treatment group. H, high-dose treatment group. ^*∗*^*p* < 0.05 between two groups. Data are expressed as means ± SEM (*n* = 10).

Because DBT appeared as the most effective herb to improve phagocytosis, oxidation, and inflammation in the in vitro experiments, it was chosen for the following study in tumor-bearing mice (Figure 4(a)). In tumor-bearing C57/BL6 mice, DBT significantly reduced the growth of inoculated tumor and the loss of body weight and gastrocnemius muscle (Figures 4(b)–4(d)) but also increased the diet intake (C group: 32.2 g/d, T group: 28.1 g/d, H group: 32.7 g/d, *p*=0.015). These effects were presented in a dose-dependent pattern. In contrast, DBT did not improve tumor growth, body weight, weight of gastrocnemius muscle, or diet intake in the nude mice group (Figures 4(b)–4(d)). These findings suggest that DBT can enhance T cell immunity to inhibit tumor growth and cancer cachexia.

### 3.5. In Vitro Anti-Inflammatory and Antioxidative Properties of DBT

**Figure 5:**
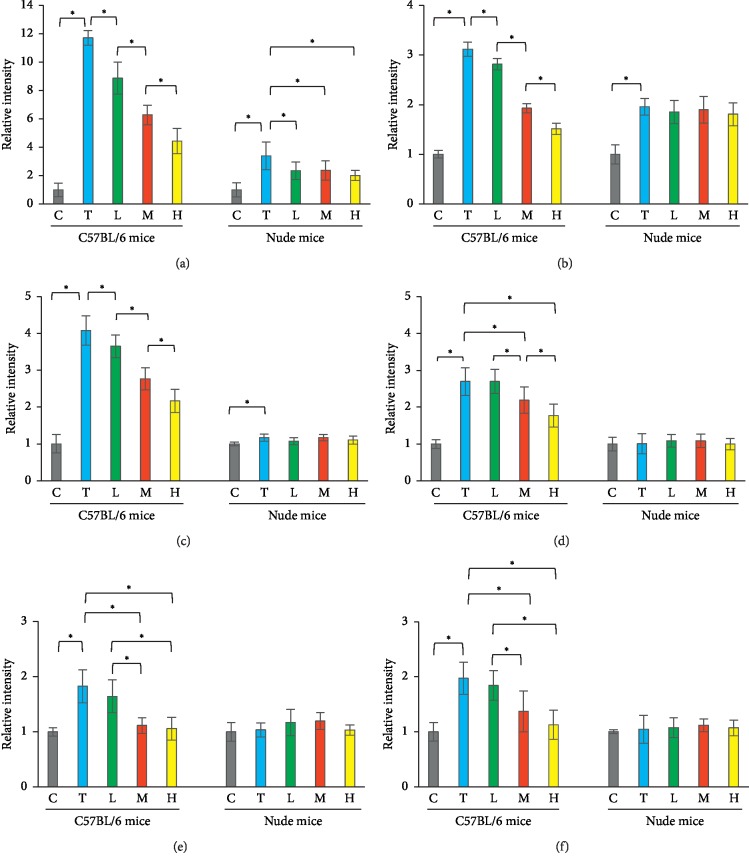
Anti-inflammatory and antioxidative abilities of the combination of *Astragalus membranaceus* and *Angelica sinensis* in tumor-bearing mice. The anti-inflammatory ability was analyzed by measuring serum IL-1*β* (a), IL-6 (b), and TNF-*α* (c) levels. The antioxidative function was evaluated by detecting serum volume of H_2_O_2_ (d), superoxide (e), and NO (f). C, control group. T, tumor group. L, low-dose treatment group. M, middle-dose treatment group. H, high-dose treatment group. ^*∗*^*p* < 0.05 between two groups. Data are expressed as means ± SEM (*n* = 10).

The serum levels of inflammatory cytokines were elevated in both C57/BL6 and nude mice after tumor inoculation (Figures 5(a)–5(c)). In C57BL/6J mice, the following DBT caused a dose-dependent reduction in serum levels of IL-1*β*, IL-6, and TNF-*α*. By contrast, in nude mice, only IL-1*β* level was suppressed by treating DBT. The serum albumin level was decreased after tumor inoculation in C57BL/6J mice, but after DBT treatment, it was elevated in a dose-dependent pattern (C group: 2.20 g/dl, T group: 1.38 g/dl, L group: 1.53 g/dl, M group: 1.75 g/dl, and H group: 1.88 g/dl, *p* < 0.001). Meanwhile, the levels of H_2_O_2_, superoxide, and NO were elevated after tumor inoculation in C57/BL6 mice, but this phenomenon was not observed in nude mice. In C57/BL6 mice, the levels of ROS and NO were subjected to a dose-dependent reduction under the DBT treatment (Figures 5(d)–5(f)). Collectively, DBT can modulate T cell immunity to attenuate cancer-related oxidation and inflammation.

### 3.6. Effects of DBT to Murine Peripheral Blood Cells and Splenocyte Differentiation

As illustrated in Table 1, tumor-bearing mice suffered from the increase of white blood cells, monocytes, and lymphocytes in peripheral bloods. DBT treatment effectively ameliorates these hematologic abnormalities in C57BL/6J mice, but its effect became modest in nude mice. This finding suggested that DBT depends on a normal T cell immunity to attenuate cancer-related leukocytosis.

**Figure 6:**
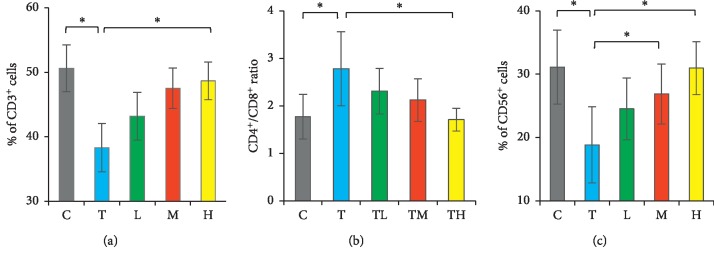
Differentiation of splenocytes was influenced by the combination of *Astragalus membranaceus* and *Angelica sinensis*. The percentage of CD3+ T (a) and CD56+ NK (c) cells and the ratio of (b) CD4+ T helper/CD8+ cytotoxic T cells in splenocytes were measured by flow cytometry. C, control group. T, tumor group. L, low-dose treatment group. M, middle-dose treatment group. H, high-dose treatment group. ^*∗*^*p* < 0.05 between two groups. Data are expressed as means ± SEM (*n* = 10).

The DBT treatment also influenced the differentiation of splenocytes (Figure 6). In C57BL/6J mice, the percentages of CD3+ T and CD56+ NK cells were decreased, but the ratio of CD4+ Th/CD8+ Tc elevated after tumor inoculation. In contrast, DBT significantly increased the percentage of T and NK cells but also recovered the Th/Tc cell ratio in a dose-dependent pattern. On the contrary, DBT treatment also tended to ameliorate cancer-induced elevation of B cells (C group: 29.2%, T group: 32.9%, L group: 32.6%, M group: 32.4%, and H group: 29.6%, *p*=0.19). These findings indicate that DBT can apply the T cell immunity to restore the differentiation of immune cells in the cancer-bearing host.

### 3.7. Effects of DBT on Th1/Th2 Cytokines and M1/M2 Polarization

**Figure 7:**
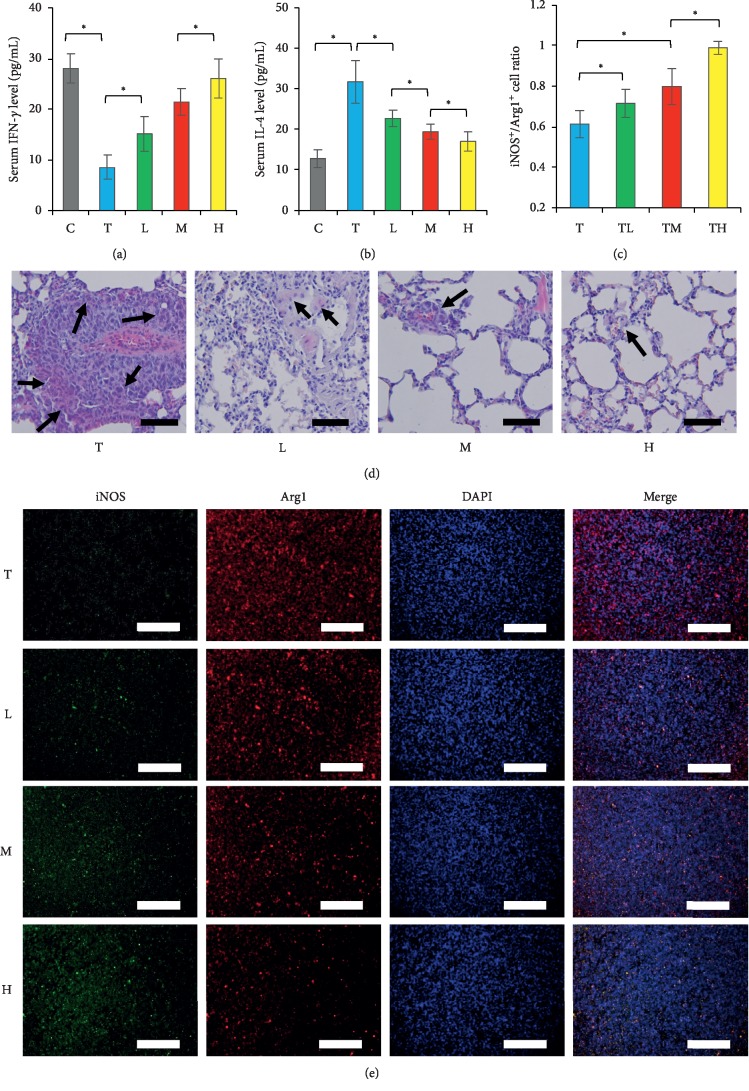
Serum levels of Th1-related IFN-*γ* (a) and Th2-related IL-4 (b) cytokines, the infiltration of tumor-associated macrophages into tumor tissue (d) (black arrows), and the polarization of iNOS+ M1- and Arg1+ M2-phenotype tumor-associated macrophages (c, e) were influenced by the combination of *Astragalus membranaceus* and *Angelica sinensis*. C, control group. T, tumor group. L, low-dose treatment group. M, middle-dose treatment group. H, high-dose treatment group. ^*∗*^*p* < 0.05 between two groups. Data are expressed as means ± SEM (*n* = 10). Scale bar: (d) 5 *μ*m; (e) 200 *μ*m.

It has been noticed that lung cancer can influence the levels of Th1/Th2 cytokines in peripheral blood and the phenotype polarization of M1/M2 macrophages in tumor microenvironment (TAMs). In clinical studies, lung cancer had been associated with the overexpression of Th2 cytokines and M2 TAMs; meanwhile, these cytokines and macrophages were correlated to a poor response of chemotherapy and a shorter survival [6, 7]. Because DBT had been shown to modulate the host immunity, we continuously explored its characteristic to the differentiation of Th cytokines and TAMs. In mice with normal immunity, the serum IFN-*γ* (a Th1 cytokine) level and the tumoral expression of Arg1+ cells (M2 TAMs) were elevated, but the IL-4 (a Th2 cytokine) level and the expression of iNOS+ cells (M1 TAMs) were diminished after tumor inoculation (Figure 7). After DBT treatment, the serum levels of IFN-*γ* and IL-4 and the tumoral expression of iNOS+ and Arg1+ cells were overturned. The effects were in a dose-dependent pattern. These results demonstrate that DBT can restore the predominance of serum Th1 cytokine and M1 TAMs in the host; therefore, it can suppress tumor growth and prolong survival in the host of malignancy.

### 3.8. Effects of DBT to Inflammatory and Angiogenic Pathways in Tumor Cells

**Figure 8:**
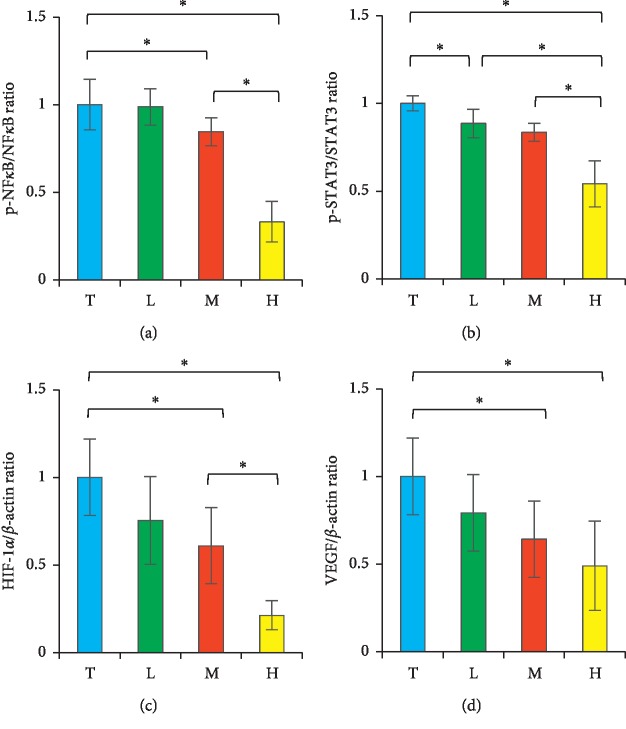
Tumoral expression of NF-*κ*B (a), STAT3 (b), HIF-1*α* (c), and VEGF (d) was regulated by the combination of *Astragalus membranaceus* and *Angelica sinensis*. The presentation of these proteins was measured by western blot. C, control group. T, tumor group. L, low-dose treatment group. M, middle-dose treatment group. H, high-dose treatment group. ^*∗*^*p* < 0.05 between two groups. Data are expressed as means ± SEM (*n* = 10).

To understand further how DBT interfered in molecular pathways of tumor cells, we examined the expression of NF-*κ*B, STAT3, HIF-1*α*, and VEGF in tumor tissues. In C57BL/6J mice, the addition of DBT can suppress expression of these given proteins. In terms of protein expression, there was a dose-dependent reduction in the DBT treatment groups when compared with the tumor group (Figure 8). As a result, DBT can downregulate the inflammation and angiogenesis pathways in the tumor-bearing mice.

## 4. Discussion

This study was to research the influence of *Astragalus membranaceus* (AM) and *Angelica sinensis* (AS) to cancer growth and cachexia in lung cancer-bearing mice. In literature, AM and AS have been shown to improve anemia, inflammation, fatigue, and the chemotherapy response in patients with lung cancer [15, 16, 25–27]. However, their direct influence to tumor cells was rarely reported. In this study, we discovered that (1) AM and AS are not toxic to normal and malignant cells. (2) AM, AS, and their combination have favorable phagocytic, antioxidative, and anti-inflammatory functions. By comparison, the combination of AM and AS at the weight ratio of 5 : 1 has the best efficacy in both in vitro and in vivo examinations. (3) The combination of AM and AS can upregulate the expression of serum Th1 cytokine, Tc cells, and M1 TAMs. It also downregulates tumoral expressions of NF-*κ*B, STAT3, HIF-1*α*, and VEGF. (4) The combination of AM and AS can suppress tumor growth and cancer cachexia in normal mice. These evidences suggest that the combination of AM and AS contains a great advantage to patients with lung cancer.

In our MTS experiments, the median lethal dose (LD50) of normal and malignant cells was not achieved even at 100 mg/ml of herbal treatment. This result is consistent to the literatures, in which the LD50 of AM and AS was about 400 mg/ml for normal cell lines, 40 and 1.6 g/kg, respectively, for normal mice [28, 29]. In the following experiments, we found that the combination of AM and AS, in a weight ratio of 5 : 1, can stimulate the strongest phagocytotic function in LPS-stimulated macrophages but also contains the best antioxidative and anti-inflammatory abilities, as compared with the single AM or AS treatment. This herbal formula is known as Danggui Buxue Tang in traditional Chinese medicine and has been used for patients with weakness or anemia for hundred years. In the modern research, Danggui Buxue Tang and its active components have shown to attenuate the release of ROS, NO, and inflammatory cytokines in animals with nonmalignant diseases [24, 26, 30, 31]. By contrast, our study pointed out its antioxidative and anti-inflammatory functions are also available in the host of malignancy. On the contrary, we also found out that its effects would become indistinct in mice with depleted T cell immunity, which strongly suggests that these functions are associated with the modulation of host immunity.

There is a variety of immune cells within tumor microenvironment. Among these cells, Th1, Tc, and NK cells and M1 TAMs are closely related to antitumoral immunity and cancer inhibition, while Th2 and M2 cells play a part in immune suppression and tumor promotion [10, 13]. With regard to the polarization of macrophages, Th1 cytokines like IFN-*γ* can promote the differentiation of M1 macrophages, while Th2 cytokines like IL-4 can stimulate of the expression of M2 phenotype. [7]. In clinic practice, the decreasing ratios of Th1/Th2 cytokines and M1/M2 TAMs are closely related to cancer progression and poor prognosis of patients [6, 7]. The ingredients of AM and AS, such as astragaloside IV, astragalus, and angelica polysaccharides, contain abilities to improve phagocytotic function of macrophages and cytotoxic activities of NK and CD8+ T cells but inhibit the functions of regulatory T cells [8, 18, 19, 32, 33]. In our research, we found that the combination of AM and AS can upregulate the serum IFN-*γ* level and the differentiation of Tc, NK cells, and M1 TAMs in tumor-bearing mice. Meanwhile, it also rebalances the ratio of Th/Tc cell ratio and suppresses the expressions of IL-2 and M2 TAMs. These findings indicate that our herbal combination has the immunomodulatory function to trigger the activation of Th1 and NK cells, the release of IFN-*γ*, the differentiation of Tc cells and M1 TAMs, and finally the inhibition of tumor growth.

During inflammation, the host immune cells typically secrete certain signaling molecules, such as tumor growth factors, angiogenic growth factors, chemokines, or cytokines, in response to a given stimulus. Of these molecules, IL-1*β*, IL-6, TNF-*α*, and IL-4 promote the phosphorylation of downstream proteins, e.g., NF-*κ*B and STAT3, which then lead to inflammation, tumor proliferation, and prevention of apoptosis in cancer [34, 35]. In our study, the combination of AM and AS enabled to inhibit production of IL-1*β*, IL-4, IL-6, and TNF-*α* and thus resulted in the inhibition of phosphorylation of NF-*κ*B and STAT3. Besides, it also enabled to impede the expression of some angiogenic molecules, such as VEGF and HIF-1*α*. The suppression of these two molecules slows down angiogenesis inside the tumor microenvironment, thus ameliorating tumor progression and distant metastasis. In addition to inhibition of inflammation and angiogenesis in the model of organ fibrosis [36], we further determined that the combined AM and AS therapy can attenuate cancer-related inflammation and tumor angiogenesis.

It has been known that free radicals are mass produced in tumor microenvironment. Bio-free radicals are generally referred to as reactive oxygen species (ROS; e.g., H_2_O_2_, superoxide, and hydroxyl radical) and reactive nitrogen species (RNS; e.g., NO). They can be produced from endogenous and exogenous sources, such as inflammation, radiation, carcinogen, and hypoxia. The excessive production of free radicals would cause oxidative stresses, which generally activate transcription factors such as NF-*κ*B, STAT3, HIF-1*α*, and AP-1 [37]. ROS and NF-*κ*B are both involved in 7,8-dihydro-8-oxoguanine (8-oxoG) and *KRAS*-mediated inflammation, oncogenesis, and the relative interaction between innate and adaptive immunity [38]. In neoplasms, inordinate NF-*κ*B promote the expression of many vital modulators of cancer progression, such as HIF-1*α*, AP-1, STAT3, and MUC1 [39]. Furthermore, the hypoxic environment of tumor tissue stimulates the release of HIF-1*α*, which upregulates the expression of vascular endothelial growth factor (VEGF) and following angiogenesis for cancer progression [40]. These indicate the close relationship between free radicals, inflammation, and oncogenesis. AM and AS have been confirmed to suppress production of free radicals and inflammation in noncancer models [30, 31]. In this study, we identified that the combination of AM and AS can scavenge free radicals in the cancer model. It has been known that persistent inflammation would aggravate carcinogenesis, local invasion, distant metastasis, and drug resistance of tumor [37]. Therefore, the strong antioxidative effect of the combination of AM and AS also contributes to its anticancer efficacy.

Tumor-promoting inflammation plays an important role in cancer cachexia. Tumor-released cytokines, such as TNF-*α* or IL-1, are involved in the NF-*κ*B and MAPK pathways to lead to breakdown of structural muscle proteins and inhibition of protein synthesis. In the murine model of chronic fatigue syndrome, the combination of AM and AS was found to increase body weight and endurance capacity and to decrease mRNA levels of IL-1*β*, TNF-*α*, NF-*κ*B, and p38MAPK [24]. In this study, we identified that it favorably modulates the loss of body weight, gastrocnemius muscle, and white adipose tissue (C group: 0.13 g, T group: 0.09 g, L group: 0.1 g, and M and H groups: 0.12 g, *p* < 0.001) in the cancer model. Consequently, the combination of AM and AS can effectively suppress cancer cachexia.

AM and AS contain numerous effective molecules. The important molecules include ferulic acid, calycosin, formononetin, Z-ligustilide, polysaccharides, and saponins. Each of these molecules features some unique functions. For example, astragalus saponins, astragalus and angelica polysaccharides, ferulic acid, and Z-ligustilide are known to suppress expression of some tumor-promoting cytokines, such as IL-1*β*, IL-6, and TNF-*α* [16, 17, 41–43]. Ferulic acid, formononetin, Z-ligustilide, astragalus saponins, and astragalus and angelica polysaccharides were reported carrying some favorable antioxidation activities [17, 30, 44–46]. Astragalus saponins and astragalus and angelica polysaccharides reportedly possess some immunomodulation functionalities [17, 32, 47]. Besides, these molecular discoveries also supported all the current findings of AM and AS which have been found in vitro or using murine models. Therefore, we conclude that the combination of AM and AS that synergizes these favorable properties should be recruited in the battle of fighting cancer.

## 5. Conclusion

In conclusion, we demonstrate here that the combined therapy of *Astragalus membranaceus* and *Angelica sinensis* can suppress oxidative stress, inflammation, tumor proliferation, and angiogenesis. In addition, this herbal combination is able to modulate the host immunity and thus inhibits cancer growth and corresponding cachexia. In the development of new anticancer agents, we consider that this herbal combination stands a good position to be reformulated to the maximum anticancer effect on the basis of the information provided herein as a short-term goal.

## Figures and Tables

**Table 1:** Effect of the combination of *Astragalus membranaceus* and *Angelica sinensis* on murine blood cell counts.

Blood cells	C57BL/6 mice					Nude mice				
	C	T	L	M	H	C	T	L	M	H
RBC (×10^6^/*μ*L)	9.3 ± 0.5	7.1 ± 1.0	7.2 ± 0.8^*∗*^	7.0 ± 1.2^*∗*^	6.9 ± 1.5^*∗*^	9.7 ± 0.6	8.5 ± 0.9^*∗*^	9.2 ± 0.6^*∗*^	9.1 ± 0.6^*∗*^	9.2 ± 0.5^*∗*#^
WBC (×10^3^/*μ*L)	11.3 ± 2.3	16.1 ± 4.4^*∗*^	12.4 ± 2.6^*∗*#^	12.0 ± 4.0^*∗*#^	11.7 ± 2.6^*∗*#^	7.5 ± 2.0	44.1 ± 1.0^*∗*^	68.6 ± 28.1^*∗*^	63.3 ± 16.8^*∗*^	57.6 ± 14.7^*∗*^
Granulocyte (×10^3^/*μ*L)	2.4 ± 0.4	2.3 ± 0.6	2.0 ± 0.8^*∗*^	2.0 ± 0.7^*∗*^	1.7 ± 0.5^*∗*^	1.8 ± 1.0	24.2 ± 9.2^*∗*^	40.1 ± 18.4^*∗*^	37.6 ± 12.0^*∗*^	39.9 ± 10.9^*∗*^
Midsized (×10^3^/*μ*L)	0.6 ± 0.1	1.0 ± 0.3^*∗*^	0.9 ± 0.3^*∗*^	0.7 ± 0.3^*∗*#^	0.7 ± 0.2^*∗*^	0.8 ± 0.3	11.4 ± 5.3^*∗*^	13.2 ± 6.2^*∗*^	12.0 ± 4.2^*∗*^	7.9 ± 4.9^*∗*^
Lymphocyte (×10^3^/*μ*L)	8.4 ± 2.1	12.9 ± 3.6^*∗*^	9.5 ± 1.9^*∗*#^	9.4 ± 3.0^*∗*#^	9.3 ± 2.0^*∗*#^	4.9 ± 1.0	16.5 ± 12.3^*∗*^	15.2 ± 5.8^*∗*^	13.8 ± 5.8^*∗*^	9.8 ± 4.3^*∗*^

The blood cell counts were measured at the sacrifice. C, control group. T, tumor alone group. L, low-dose treatment group. M, medium-dose treatment group. H, high-dose treatment group. ^#^*p* < 0.05 as compared to the C group. ^*∗*^*p* < 0.05 as compared to the T group. Data are expressed as means ± SEM (*n* = 10).

## Data Availability

The data used to support the findings of this study are available from the corresponding author upon request.

## Abbreviations

2-OH-E+: 2-Hydroxyethidium
AM: *Astragalus membranaceus*
ANOVA: One-way analysis of variance
APS: Astragalus polysaccharides
AS: *Angelica sinensis*
AS-IV: Astragaloside IV
AST: Astragalus saponin
BHK: Baby hamster kidney cell
DAF-2/DA: 4,5-Diaminofluorescein diacetate
DAF-2T: Diaminofluorescein-triazole
DBT: Danggui Buxue Tang
DCF: 2′,7′-Dichlorofluorescein
DCFH-DA: Dichlorofluorescin diacetate
DHE: Dihydroethidium
DMEM: Dulbecco's modified eagle's medium
DPPH: Ethanolic 2,2-diphenyl-1-picrylhydrazyl
ELISA: Enzyme-linked immunosorbent assay
FBS: Fetal bovine serum
FITC: Fluorescein isothiocyanate
GSH: Glutathione
HIF-1*α*: Hypoxia-inducible factor 1-alpha
IF: Immunofluorescence
IL-1: Interleukin-1
INF-*γ*: Interferon-*γ*
LLC: Lewis lung carcinoma cell
LPS: Lipopolysaccharide
MTS: 3-(4,5-Dimethylthiazol-2-yl)-5-(3-carboxymethoxyphenyl)-2-(4-sulfophenyl)-2H-tetrazolium
NF-*κ*B: Nuclear factor kappa-light-chain-enhancer of activated B cells
NK: Natural killer cell
NO: Nitric oxide
PBS: Phosphate-buffered saline
PE: Phycoerythrin
ROS: Reactive oxygen species
SEM: Standard error of mean
SDS-PAGE: Sodium dodecyl sulfate polyacrylamide gel electrophoresis
STAT3: Signal transducer and activator of transcription 3
TAM: Tumor-associated macrophage
Tc: Cytotoxic T cell
Th: T helper cell
Th1: T helper 1 cell
TNF-*α*: Tumor necrosis factor-*α*
UPLC: Ultraperformance liquid chromatography
VEGF: Vascular endothelial growth factor.
